# Rosuvastatin protects against coronary microembolization-induced cardiac injury via inhibiting NLRP3 inflammasome activation

**DOI:** 10.1038/s41419-021-03389-1

**Published:** 2021-01-12

**Authors:** Ao Chen, Zhangwei Chen, You Zhou, Yuan Wu, Yan Xia, Danbo Lu, Mengkang Fan, Su Li, Jinxiang Chen, Aijun Sun, Yunzeng Zou, Juying Qian, Junbo Ge

**Affiliations:** Department of Cardiology, Zhongshan Hospital, Fudan University; Shanghai Institute of Cardiovascular Diseases; National Clinical Research Center for Interventional Medicine, Shanghai, China

**Keywords:** Cell death, Cardiovascular diseases

## Abstract

Coronary microembolization (CME), a common reason for periprocedural myocardial infarction (PMI), bears very important prognostic implications. However, the molecular mechanisms related to CME remain largely elusive. Statins have been shown to prevent PMI, but the underlying mechanism has not been identified. Here, we examine whether the NLRP3 inflammasome contributes to CME-induced cardiac injury and investigate the effects of statin therapy on CME. In vivo study, mice with CME were treated with 40 mg/kg/d rosuvastatin (RVS) orally or a selective NLRP3 inflammasome inhibitor MCC950 intraperitoneally (20 mg/kg/d). Mice treated with MCC950 and RVS showed improved cardiac contractile function and morphological changes, diminished fibrosis and microinfarct size, and reduced serum lactate dehydrogenase (LDH) level. Mechanistically, RVS decreased the expression of NLRP3, caspase-1, interleukin-1β, and Gasdermin D N-terminal domains. Proteomics analysis revealed that RVS restored the energy metabolism and oxidative phosphorylation in CME. Furthermore, reduced reactive oxygen species (ROS) level and alleviated mitochondrial damage were observed in RVS-treated mice. In vitro study, RVS inhibited the activation of NLRP3 inflammasome induced by tumor necrosis factor α plus hypoxia in H9c2 cells. Meanwhile, the pyroptosis was also suppressed by RVS, indicated by the increased cell viability, decreased LDH and propidium iodide uptake in H9c2 cells. RVS also reduced the level of mitochondrial ROS generation in vitro. Our results indicate the NLRP3 inflammasome-dependent cardiac pyroptosis plays an important role in CME-induced cardiac injury and its inhibitor exerts cardioprotective effect following CME. We also uncover the anti-pyroptosis role of RVS in CME, which is associated with regulating mitochondrial ROS.

## Introduction

Coronary microcirculation performs a vital role in maintaining appropriate myocardial perfusion. Apart from epicardial coronary arteries obstruction, coronary microvascular dysfunction has also been confirmed as an important mechanism of myocardial ischemia^1^. Coronary atherosclerotic plaque debris derived from spontaneous plaque rupture or iatrogenic rupture during the percutaneous coronary intervention (PCI) are able to block the coronary microcirculation, leading to coronary microembolization (CME), which serves as a considerable cause of coronary microvascular dysfunction^2,3^. The accumulated evidence suggests that CME bears very important prognostic implications^4–6^, discovering approaches for alleviating CME-induced myocardial injury are therefore urgently required.

There are strong experimental data indicating that CME causes patchy microinfarcts with a subsequent inflammatory reaction, which results in contractile dysfunction^7,8^. The NOD-like receptor pyrin containing 3 (NLRP3) inflammasome, an intracellular protein complex activated upon tissue injury, consists of NLRP3, apoptosis-associated speck-like protein containing a CARD (ASC) and caspase-1. On activation, caspase-1 induces interleukin (IL)-1β activation by cleaving pro-IL-1β to its active form, triggering and amplifying sterile inflammatory responses. Strikingly, Gasdermin D (GSDMD) has also been demonstrated to be cleaved by caspase-1. The N-terminal domains (GSDMD-N) perforate the membrane and induce pyroptosis, a new type of inflammatory programmed necrosis. Previous evidence implies an important role for the NLRP3 inflammasome in the pathogenesis of several cardiovascular diseases, such as atherosclerosis, atrial fibrillation and myocardial infarction^9–11^. We have previously reported that the activation of the NLRP3 inflammasome can be triggered by tumor necrosis factor (TNF)-α and hypoxia stimulation, a condition mimics the ischemic micro-environment induced by CME^12^. However, the status of the NLRP3 inflammasome activation following CME in vivo has not been investigated.

Apart from cholesterol-lowering effects, statins exert multiple beneficial effects on the cardiovascular system include antioxidant effects, stabilizing atherosclerotic plaques, repressing inflammatory reaction and improving the function of several different cell types^13,14^. Recent studies demonstrated that statins could also suppress the activation of the NLRP3 inflammasome^15–17^. Intriguingly, clinical trials revealed significant improvement of coronary flow reserve in patients with coronary microvascular dysfunction following statins treatment, therefore, statins have been recommended to treat coronary microvascular dysfunction^18,19^. However, the underlying mechanisms have not been fully understood.

In the present study, mice CME model was established to investigate the role of the NLRP3 inflammasome in cardiac dysfunction following CME and to unveil the effects of rosuvastatin (RVS) on the activation of the NLRP3 inflammasome.

## Results

### NLRP3 expression was increased in cardiac tissues from mice with CME

**Fig. 1:**
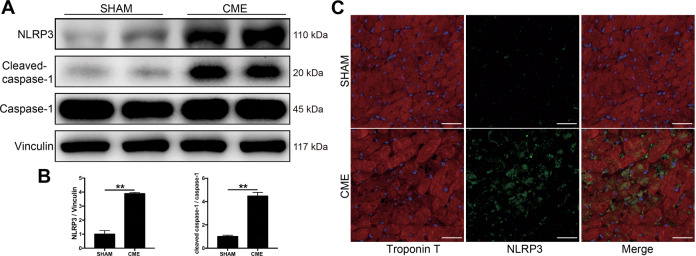
NLRP3 and cleaved-caspase-1 are upregulated in mice heart 3 days after CME intervention. **A** Representative immunoblots of NLRP3 and cleaved-caspase-1 expression in sham and CME mice hearts. **B** Densitometric analysis of relative protein expressions, Vinculin is used as loading control. *n* = 4 per group. Data represent the mean ± SEM of three replicates. **C** Representative images of immunofluorescent double staining of Troponin T and NLRP3 in cardiac tissues. Scale bars = 50 µm. *n* = 4 per group. ^∗^*P* < 0.05, ^∗∗^*P* < 0.01.

To investigate the potential role of the NLRP3 inflammasome in the pathophysiological process of CME, the key protein NLRP3 and caspase-1 were firstly examined in mice hearts 3 days after CME establishment. Our results demonstrated that NLRP3 expression was significantly upregulated in the CME hearts compared to sham hearts (Figs. 1A, B and S1). Furthermore, the expression of cleaved-caspase-1, the activated form of caspase-1, was also pronouncedly increased following CME, indicating the assembly of the NLRP3 inflammasome in mice with CME (Fig. 1A, B). Immunofluorescence double staining of Troponin T and NLRP3 furnished evidence that the expression of NLRP3 was upregulated following CME and primarily located in cardiomyocytes (Fig. 1C). In addition, we observed that CME mice hearts exhibited a significant increase in the expression level of IL-1β (Fig. S1), a key inflammatory factor downstream of the NLRP3 inflammasome. These findings hinted a strong correlation between the NLRP3 inflammasome activation and CME pathogenesis.

### Inhibiting NLRP3 inflammasome by MCC950 attenuated cardiac dysfunction and injury following CME

**Fig. 2:**
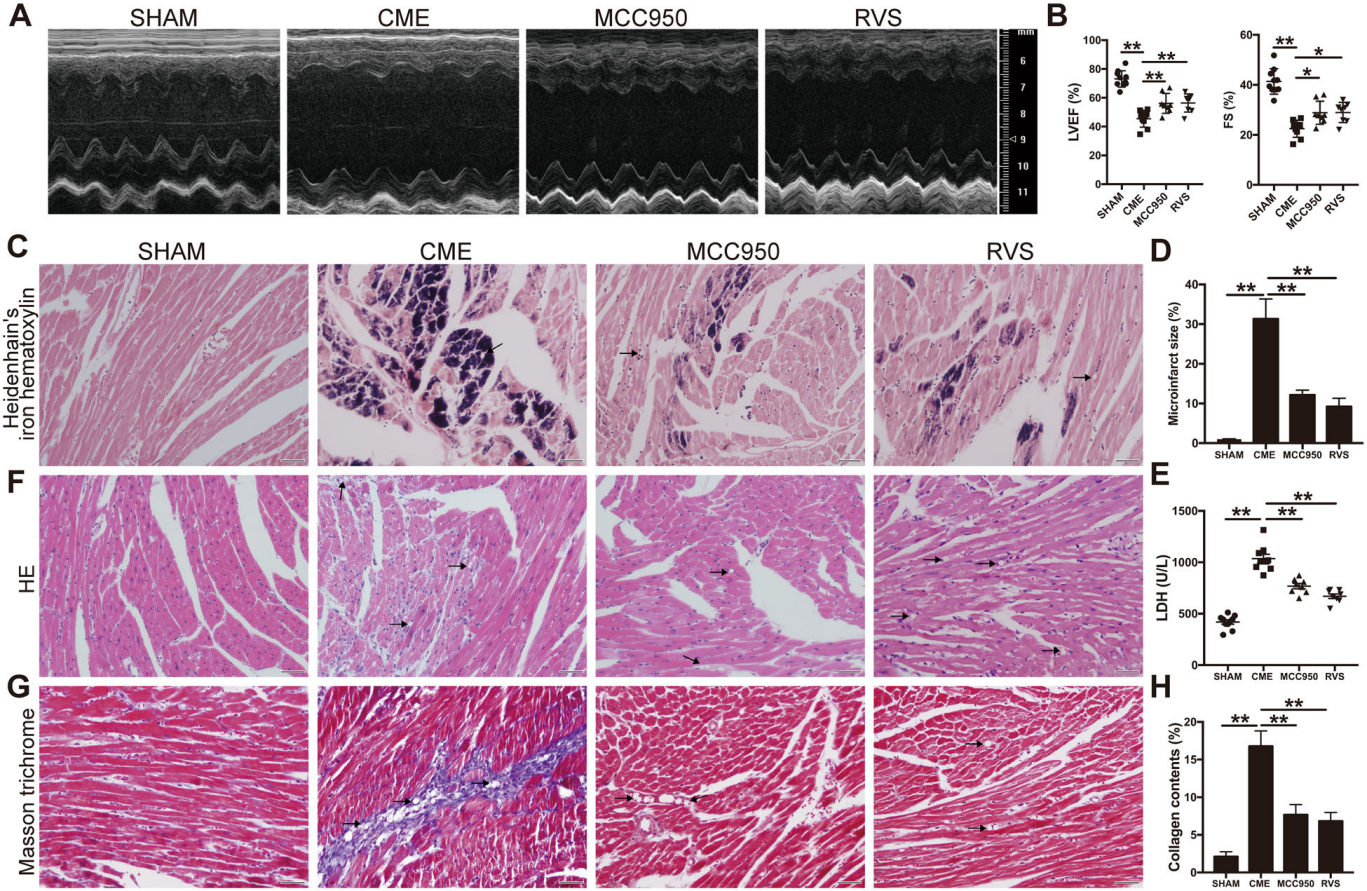
MCC950 and RVS protect against CME-induced cardiac dysfunction and injury. **A** Representative M-mode echocardiograms for each group at 3 days after CME intervention. Mice were treated with RVS and the selective NLRP3 inflammasome inhibitor MCC950 three days prior and after CME intervention at 40 and 20 mg/kg/d respectively. **B** Left ventricular ejection fraction (LVEF) and fractional shortening (FS) are measured using Doppler echocardiography. *n* = 8 to 10 per group. Data represent the mean ± SEM. **C** Representative images of Heidenhain’s iron hematoxylin staining for each group to visualize microinfarct areas, the microinfarct areas are stained into dark gray. **D** The quantitative analysis of microinfarct areas. *n* = 8 to 10 per group. The quantification is representative of the percentage of microinfarct area in 5 fields ± SEM by random. **E** Serum lactate dehydrogenase (LDH) levels are measured in each group. *n* = 8 to 10 per group. Data represent the mean ± SEM. **F** Representative images of HE staining to visualize the local micro-infracted lesions for each group. **G** Representative images of Masson trichrome staining of the ventricular sections of each group. **H** The quantitative analysis of collagen contents. *n* = 8 to 10 per group. The quantification is representative of the percentage of collagen contents in 5 fields ± SEM by random. Scale bars = 50 µm. Black arrows indicate the microspheres. ^∗^*P* < 0.05, ^∗∗^*P* < 0.01.

Transthoracic echocardiography was performed to evaluate cardiac function 3 days after the establishment of CME. Echocardiography results showed a significant decrease of left ventricular ejection fraction (LVEF) and fractional shortening (FS) in mice with CME (Fig. 2A, B). We also observed a remarkable increase of microinfarct size surrounding the microspheres in CME heart tissues as shown by Heidenhain’s iron hematoxylin staining (Fig. 2C, D). Notably, the lactate dehydrogenase (LDH) in the serum of mice subjected to CME are at significantly higher levels as compared to sham surgery (Fig. 2E). In addition, the CME heart tissues were disorganized and stained strongly for total collagen as evidenced by HE and Masson trichrome staining (Fig. 2F–H). These results indicated certain cardiac damage following CME. To evaluate the functional significance of the NLRP3 inflammasome in CME-induced cardiac injury, we used the selective inhibitor MCC950 at 20 mg/kg/d to block the NLRP3 inflammasome in mice with CME. In the setting of the blunted NLRP3 inflammasome, the cardiac contractile function was improved remarkably, as demonstrated by increased LVEF and FS in MCC950-treated mice (Fig. 2A, B). Meanwhile, mice treated with MCC950 showed notably decreased serum LDH levels compared with CME controls (Fig. 2E). Furthermore, cardiac tissue staining revealed shrinking microinfarct size, improved tissue arrangement and reduced collagen deposition with the treatment of MCC950 in mice with CME (Fig. 2C, D, F–H). Taken together, our results indicated an important role for the NLRP3 inflammasome in CME-induced cardiac injury.

### RVS protected against CME-induced cardiac dysfunction and injury

To explore the effects of statin on CME-induced cardiac injury, mice exposed to CME were pretreated with RVS at 40 mg/kg/d. We observed that treatment with RVS significantly ameliorated cardiac contractile dysfunction in response to CME (Fig. 2A, B) and normalized levels of LDH in serum (Fig. 2E). Consistent with the results of MCC950 treatment, prominently decreased microinfarct size and collagen deposition, attenuated myocardial disorganization were observed in hearts of RVS-treated mice (Fig. 2C, D, F–H). These results provided strong evidence supporting the protective effect of RVS in CME and associated pathologies.

### RVS restrained the activation of NLRP3 inflammasome in mice with CME

**Fig. 3:**
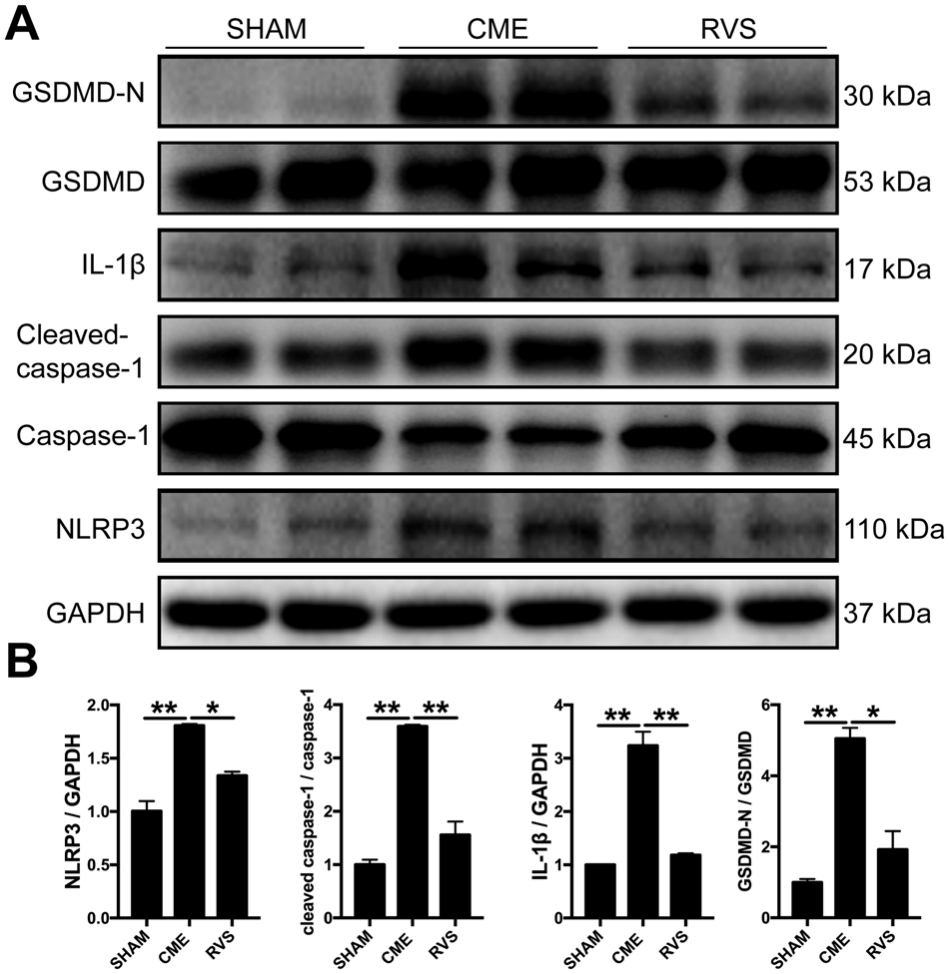
RVS restrains the activation of the NLRP3 inflammasome at 3 days after CME intervention. **A** Representative immunoblots for myocardial NLRP3, caspase-1, cleaved-caspase-1, IL-1β, GSDMD, GSDMD-N in the hearts of sham, CME and RVS-treated mice. **B** The densitometric analysis of relative protein expressions, GAPDH is used as loading control. *n* = 4 per group. Data represent the mean ± SEM of three replicates. ^∗^*P* < 0.05, ^∗∗^*P* < 0.01.

Three days after CME induction and the drug intervention, the mice were sacrificed and the molecular events underlying the effects of RVS were further investigated. Western blotting results suggested that the levels of NLRP3 and the downstream proteins cleaved-caspase-1, IL-1β were notably decreased after RVS treatment (Fig. 3A, B). Myocardial pyroptosis following NLRP3 inflammasome activation critically contributes to cardiac damage. The further assessment indicated that RVS treatment effectively inhibited the cleavage of GSDMD to the active form GSDMD-N (Fig. 3A, B), which dominates the process of pyroptosis. Therefore, these data suggested that the protective role of RVS in CME possibly depended on the restraint of the NLRP3 inflammasome and pyroptosis.

### Caspase-1 inhibitor and RVS prevented TNF-α plus hypoxia-induced pyroptosis in H9c2 cells

**Fig. 4:**
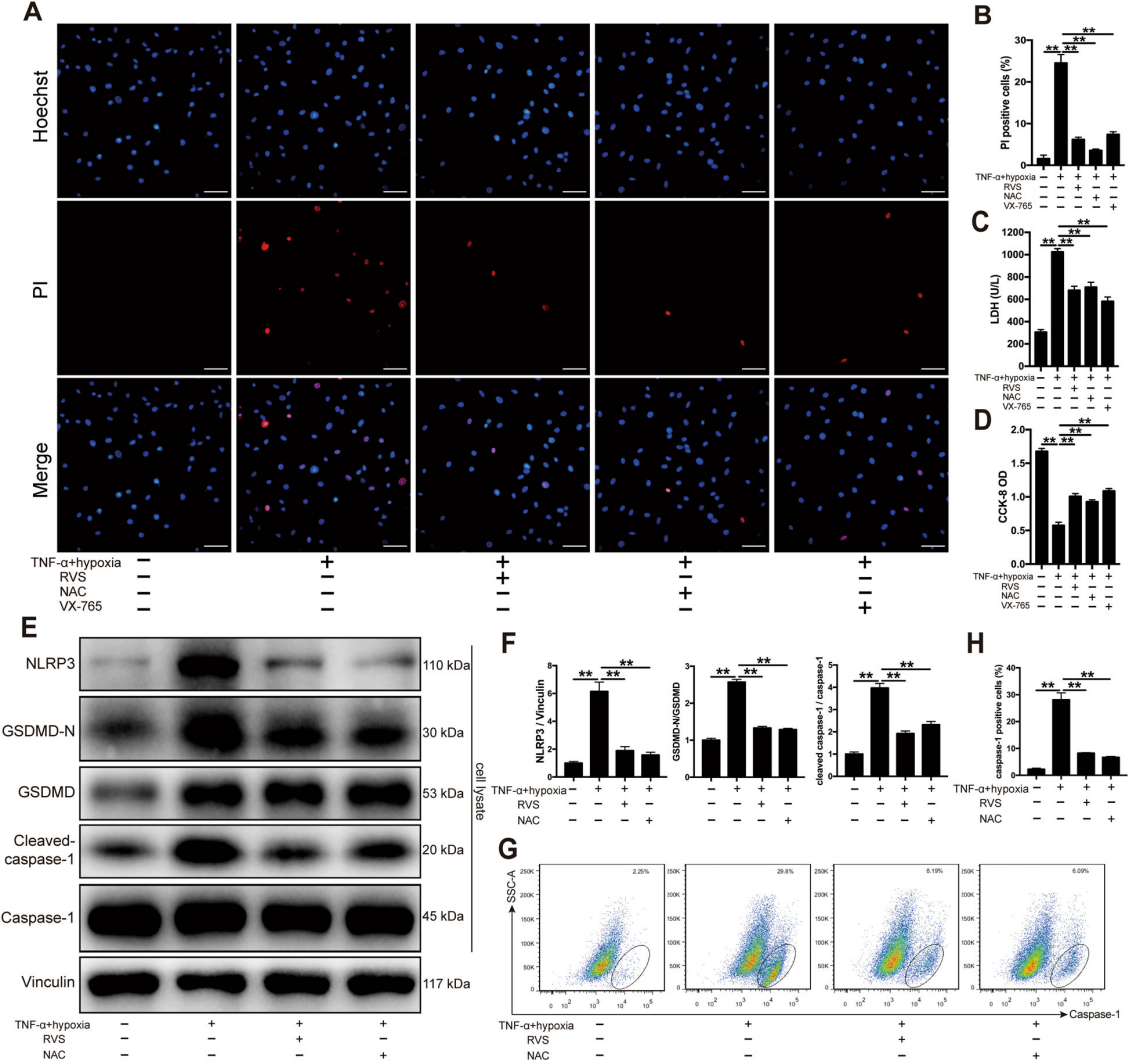
RVS and ROS scavenger inhibit the activation of the NLRP3 inflammasome and pyroptosis in H9c2 cells. **A** The H9c2 cells are stimulated with 40 ng/ml TNF-α combined with hypoxia for 12 h to active the NLRP3 inflammasome. The caspase-1 selective inhibitor VX-765 and ROS scavenger N-acetylcysteine (NAC) are added 2 h before the stimulation at 20 μM and 5 mM respectively. The pyroptotic cells are determined by Hoechst 33342/PI staining, wherein the nuclei are stained to blue by Hoechst 33342, and the pyroptotic cells are stained to red by PI. Scale bars = 50 μm. **B** The quantitative analysis of PI positive cells. *n* = 6 per group. The quantification is representative of the percentage of pyroptotic cells in 5 fields ± SEM by random. **C** LDH release is measured by a cytotoxicity detection LDH kit. *n* = 6 per group. **D** Cell viability is measured by a CCK-8 kit, and the optical densities (ODs) are compared among the groups. *n* = 6 per group. **E** Representative immunoblots for NLRP3, caspase-1, cleaved-caspase-1, GSDMD, GSDMD-N in H9c2 cells. **F** The densitometric analysis of relative protein expressions. Vinculin is used as a loading control. *n* = 4 per group. **G** Cells with active caspase-1 in each group are stained with FLICA (FAM-YVAD-FMK) probe and detected using flow cytometer. **H** The percentages of caspase-1 positive cells are compared among the groups. *n* = 6 per group. Data represent the mean ± SEM. All experiments were repeated three times. ^∗^*P* < 0.05, ^∗∗^*P* < 0.01.

To gain an in-depth understanding of the role of statin in the regulation of NLRP3 inflammasome-dependent pyroptosis during CME, TNF-α plus hypoxia were used to simulate the ischemic micro-environment induced by CME in H9c2 cardiomyoblasts. The levels of cellular injury were examined to evaluate pyroptotic cell death. Hoechst 33342/propidium iodide (PI) fluorescent staining revealed that stimulation with 40 ng/ml TNF-α combined with hypoxia for 12 h markedly increased the PI uptake levels in H9c2 cells (Fig. 4A, B). Meanwhile, we also observed increased LDH level and decreased cell viability level under the condition of TNF-α and hypoxia (Fig. 4C, D). Furthermore, TNF-α plus hypoxia significantly increased the expression of NLRP3, cleaved-caspase-1 and GSDMD-N (Fig. 4E, F). Flow cytometry analysis further revealed a distinct increase of caspase-1 activity in H9c2 cells (Fig. 4G, H), suggesting the activation of the NLRP3 inflammasome. At the concentration of 20 μM, caspase-1 inhibitor VX-765 largely rescued TNF-α and hypoxia-induced pyroptotic cell death (Fig. 4A–D), demonstrating the key role of caspase-1 in the death of H9c2 cells. Meanwhile, we found RVS evidently diminished caspase-1 activity and suppressed the activation of NLRP3 inflammasome in H9c2 cells (Fig. 4E–H). As expected, the pyroptotic cell death in H9c2 cells exposed to TNF-α and hypoxia was markedly reversed by RVS (Fig. 4A–D). These data suggested RVS prevented TNF-α plus hypoxia-induced pyroptosis possibly by inhibiting the activation of NLRP3 inflammasome in H9c2 cells.

### ROS scavenger prevented TNF-α plus hypoxia-induced pyroptosis and caspase-1 activation in H9c2 cells

It was well established that the NLRP3 inflammasome can be activated by danger signals, including reactive oxygen species (ROS)^20^. To examine the role of ROS in TNF-α plus hypoxia-induced pyroptosis, H9c2 cells were pretreated with the ROS scavenger N-acetylcysteine (NAC) at 5 mM. As described in Fig. 4A–D, NAC alone was sufficient to prevent TNF-α plus hypoxia-induced cell death. Further immunoblotting and flow cytometry analysis showed that NAC treatment blunted the increase in the cleavage of GSDMD and caspase-1 activity in a fashion similar to that of RVS (Fig. 4E–H). Accordingly, ROS production contributed to the pyroptotic death in H9c2 cells exposed to TNF-α and hypoxia.

### RVS restored the energy metabolism and oxidative phosphorylation in CME

**Fig. 5:**
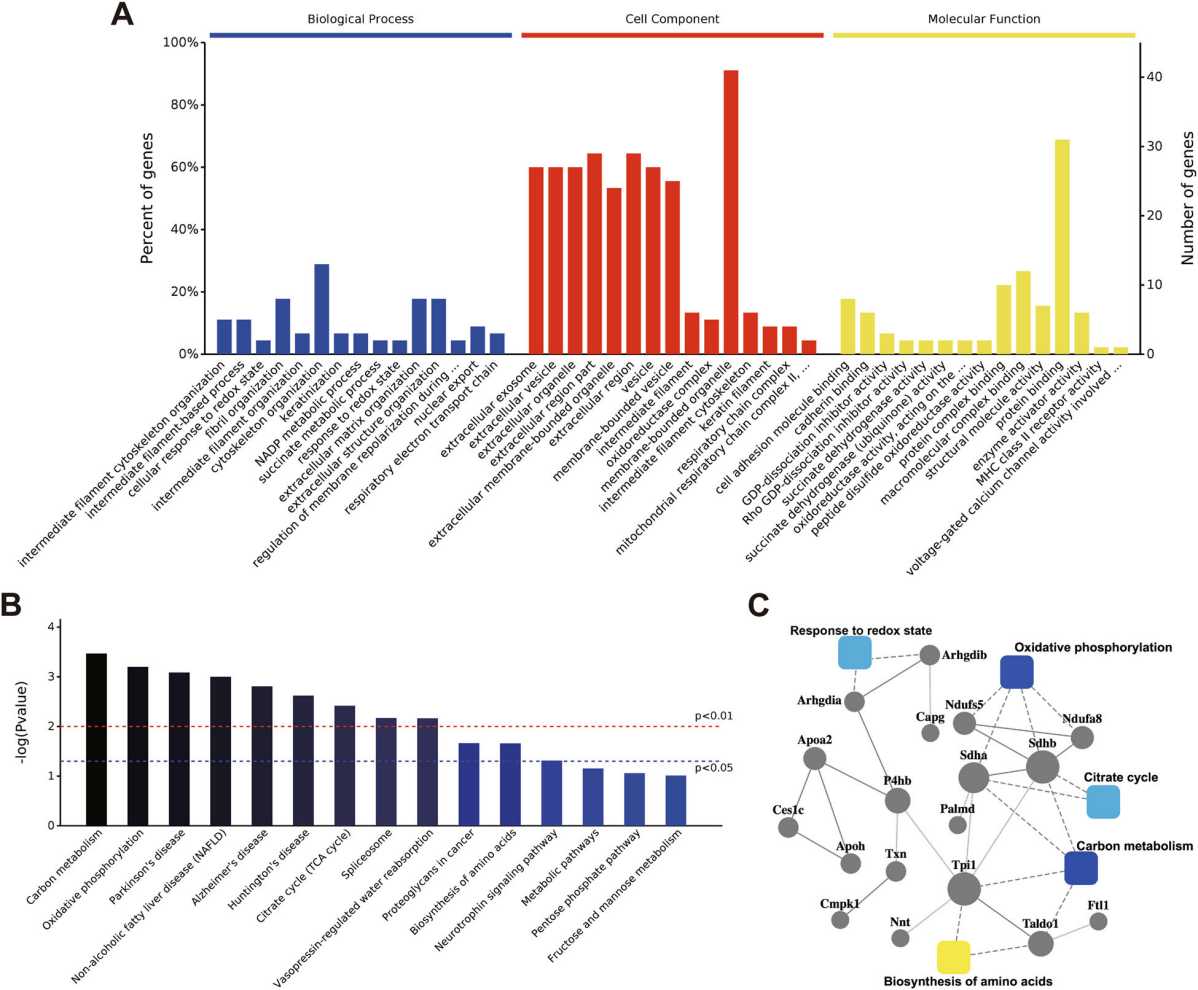
Proteomics and bioinformatics analysis of the proteins reversed by RVS, RVS restores the energy metabolism and oxidative phosphorylation in CME. **A** Bar chart depicting the GO classification of the 63 differentially expressed proteins in terms of biological process, cellular component and molecular function. **B** Top 15 KEGG pathways enriched by the differentially expressed proteins. **C** PPI analysis of the energy metabolism-related pathways, including carbon metabolism, oxidative phosphorylation, citrate cycle and response to redox state, wherein SDHA and SDHB carried the heaviest weight.

To get insight into the mechanism by which RVS inhibits the NLRP3 inflammasome, proteomics analysis was performed to discover the proteins rescued by RVS in mice with CME. Bioinformatics analysis revealed that 63 proteins reached statistical significance among the sham, CME and RVS-treated mice, indicating that these 63 proteins were upregulated or downregulated in mice with CME compared with the sham group, and then were reversed by RVS treatment. GO enrichment analysis demonstrated that the 63 differentially expressed proteins mainly located in membrane-bounded intracellular organelles or respiratory chain complex, and participated in several metabolic processes or response to redox state (Fig. 5A). Energy metabolism and oxidative phosphorylation-related pathways were also significantly enriched in the Kyoto Encyclopedia of Genes and Genome (KEGG) pathway analysis (Fig. 5B). PPI analysis provided us with distinct networks to get insight into the proteins and physiological processes which were reversed by RVS in mice with CME. Succinate dehydrogenase (SDH), subunit A and SDHB are important components of both the citrate cycle and the mitochondrial respiratory chain^21^. As shown in Fig. 5C, SDHA and SDHB carried the heaviest weight in the PPI analysis. These data provided us with the evidence that RVS restored the impairment of mitochondrial function induced by CME.

### RVS alleviated the disruption of mitochondria and the production of ROS

**Fig. 6:**
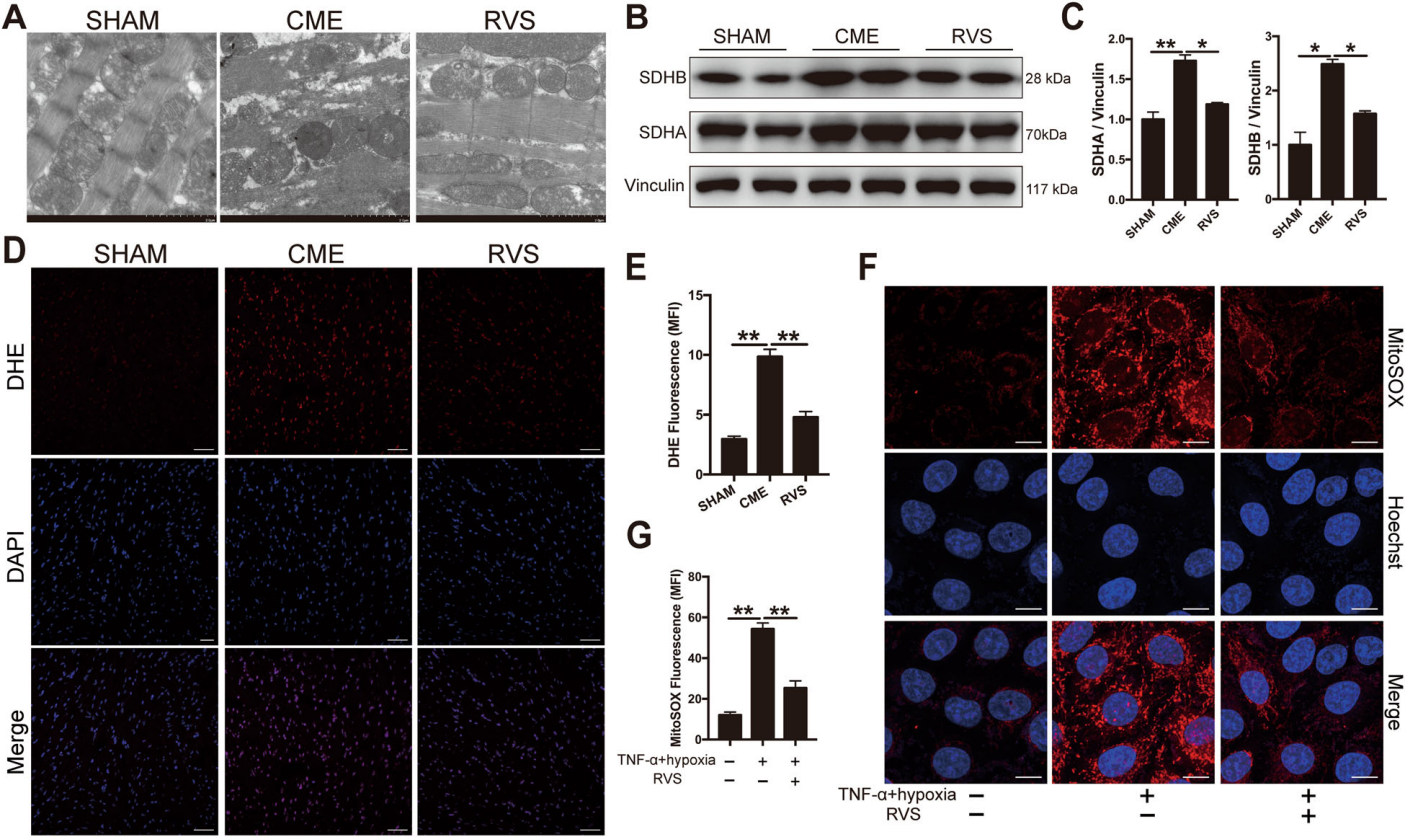
RVS alleviates the damage of mitochondria and the production of ROS. **A** Representative mitochondrial photographs, detected by transmission electron microscopy. Scale bars = 2 μm. **B** Representative immunoblots of SDHA and SDHB expression in the cardiac tissues of sham, CME and RVS-treated mice. **C** The densitometric analysis of relative protein expressions. Vinculin is used as loading control. *n* = 4 per group. Data represent the mean ± SEM of three replicates. **D** Representative fluorescence images of ROS staining using dihydroethidium (DHE) in heart tissues (blue, DAPI-stained nuclei; red, DHE-stained ROS). Scale bars = 50 μm. **E** The quantitative analysis of DHE intensity. *n* = 4 per group. The quantification is representative of MFI in 5 fields ± SEM by random. **F** Cellular mitochondrial ROS are stained with MitoSOX probe. Scale bars = 10 μm. **G** The quantitative analysis of MitoSOX MFI. *n* = 6 per group. The quantification is representative of MFI in 5 fields ± SEM by random. ^∗^*P* < 0.05, ^∗∗^*P* < 0.01.

To verify the damage of mitochondria, CME heart tissues were scanned by transmission electron microscopy (TEM). TEM revealed that CME increased the number of vacuolated and malformed mitochondria and damaged their crista integrity, which were significantly rescued by RVS (Fig. 6A). Immunoblotting analysis revealed that treatment with RVS reduced the increase of SDHA and SDHB induced by CME, further demonstrating the protective role of RVS in mitochondrial dysfunction following CME (Fig. 6B, C). It has been recognized that the disruption of mitochondria and oxidative phosphorylation is linked to the production of mitochondrial ROS^22^. Therefore, we further evaluated ROS levels by measuring mean fluorescence intensity (MFI) of fluorescent staining of ROS. Dihydroethidium (DHE) staining of myocardial slices indicated that RVS dramatically suppressed the outburst of ROS production induced by CME (Fig. 6D, E). Next, we also evaluated the mitochondrial ROS levels in vitro using MitoSOX probe. The presence of TNF-α plus hypoxia led to a much higher level of mitochondrial ROS production in H9c2 cells, which was diminished by RVS treatment (Fig. 6F, G). Combined, these results showed that RVS alleviated the disruption of mitochondria and decreased the production of ROS following CME, which might be the underlying mechanism of the anti-pyroptosis function of RVS.

## Discussion

The present study demonstrated that the NLRP3 inflammasome was activated in the mice heart subjected to CME. We observed that CME-induced significant cardiac contractile dysfunction, myocardial fibrosis and injury. To further explore the role of the NLRP3 inflammasome in the cardiac injury, mice with CME were pretreated with the specific inhibitor MCC950. Decreased myocardial fibrosis and injury, improved cardiac contractile function were evidenced in MCC950-treated mice. Meanwhile, mice with CME exposed to RVS also exhibited prominently decreased microinfarct size and ameliorated cardiac contractile dysfunction. Various studies suggested that ROS are key factors contributing to the activation of the NLRP3 inflammasome^23–25^. ROS scavenger NAC was verified to inhibit NLRP3 inflammasome-dependent pyroptosis in H9c2 cells in the present study. Further proteomics analysis unraveled an important finding that energy metabolism and oxidative phosphorylation-related processes were disordered in CME hearts, and these abnormal alterations were rescued by RVS. The generation of ROS is closely related to mitochondrial function and oxidative phosphorylation. TEM and ROS analysis further revealed that RVS significantly restored CME-induced mitochondrial damage and ROS production. Consistent results were obtained by using H9c2 cells stimulated with TNF-α and hypoxia. These results suggested that the regulation of mitochondrial function, ROS production and subsequent activation of the NLRP3 inflammasome might be involved in the cardioprotective effect of RVS in CME.

The prevailing paradigm suggests that inflammatory response, cardiomyocyte apoptosis and oxidation of contractile proteins contribute to the CME-induced myocardial injury and contractile dysfunction^7,26,27^, however the specific underlying mechanisms remain largely elusive. The NLRP3 inflammasome is a macromolecular structure responsible for sensing danger and triggering a local or systemic inflammatory response. When activated, the NLRP3 inflammasome produces large amounts of active inflammatory factors such as IL-1β and IL-18^10^. Numerous studies have highlighted the crucial role of the NLRP3 inflammasome in several cardiovascular diseases, including myocardial infarction^10^, myocardial ischemia-reperfusion injury^28^, heart failure^29^, atrial fibrillation^11^, and hypertension^30^. The previous study has reported that the selective NLRP3 inflammasome inhibitor MCC950 reduces infarct size and preserves cardiac function in a randomized, blinded translational large animal myocardial infarction model^31^. The Canakinumab Anti-inflammatory Thrombosis Outcome Study trial also provides strong support for the hypothesis that the NLRP3 inflammasome pathway contributes to the pathogenesis of the atherosclerotic disease, demonstrating diverse clinical benefits of inhibiting IL-1β for cardiovascular events^32^.

Intriguingly, beyond triggering inflammation, the NLRP3 inflammasome-dependent pyroptosis has also been brought to the forefront^33^. Previous studies have demonstrated that the NLRP3 inflammasome locates in diverse cardiac cells, such as cardiomyocytes, fibroblasts, endothelial cells and macrophages^28,34–37^. Importantly, the NLRP3 inflammasome may have cell-intrinsic roles in the different cell types. Activation of the NLRP3 inflammasome promotes processing and release of IL-1β and the subsequent initial inflammation in cardiac fibroblasts and macrophages, whereas it induces caspase-1-dependent pyroptosis in cardiomyocytes^38^. In the present study, we focused on the loss of cardiomyocytes induced by pyroptotic cell death following CME. We explored that the NLRP3 inflammasome was significantly activated in CME mice hearts, accompanied by the increases of IL-1β and GSDMD-N. Further immunofluorescence double staining of Troponin T and NLRP3 demonstrated the expression of NLRP3 was primarily located in cardiomyocytes. Meanwhile, we observed obvious myocardial injury in mice with CME, prompting a relationship between the NLRP3 inflammasome and CME pathogenesis. This finding was further strengthened by the study that inhibiting the NLRP3 inflammasome by MCC950 attenuated cardiac dysfunction, decreased myocardial microinfarct size and injury. In vitro, TNF-α and hypoxia efficiently induced the activation of the NLRP3 inflammasome and pyroptosis in H9c2 cells. These observations strengthened our notion that the activation of the NLRP3 inflammasome pathway contributed to the myocardial injury in CME.

The beneficial cardiovascular pleiotropic effects of statins, such as anti-oxidant effects and anti-inflammatory effects, have drawn our attention for years^13^, giving us clues regarding the effective role of statins in CME. In support of this notion, clinical evidence has reported a reduced incidence of periprocedural myocardial injury, lower microvascular resistance index and a better outcome in patients given statin treatment when undergoing PCI^39^. Interestingly, protective role still exists when statin therapy is initiated after PCI^40^. The inhibition of myocardial inflammation and apoptosis in CME animal models by statin treatment has also been reported^41,42^. Consistent with these findings, here we reported that RVS exerted cardioprotective role in mice with CME, which was supported by the observation that mice pretreated with RVS exhibited improved cardiac function and alleviated myocardial injury following CME. Considering that the contribution of the NLRP3 inflammasome varies among different cardiac cells, the effects of RVS on the NLRP3 inflammasome may also differ. While majority previous studies focused on the inflammation suppression effect of RVS via inhibiting the NLRP3 inflammasome^43–45^, relatively little is known about the anti-pyroptosis role of RVS, especially in cardiomyocytes. In our study, RVS treatment suppressed the expression of cleaved-caspase-1 and IL-1β, as well as GSDMD-N, implying the inhibitory role of RVS in the NLRP3 inflammasome pathway. These effects of RVS were further confirmed by carrying out cell line studies on H9c2 cardiomyoblasts. Therefore, we provided a novel mechanism highlighting that RVS protected against CME-induced cardiac injury possibly through inhibiting NLRP3 inflammasome activation and pyroptosis.

Metabolic reprogramming has been reported to play a critical role in NLRP3 inflammasome activation. The NLRP3 inflammasome has been shown to sense diverse metabolites and is regulated by components of glycolysis^46^. Importantly, there is also clearly a damaging association between NLRP3 inflammasome activation and mitochondrial dysregulation^47^. The major role of mitochondria is the efficient coupling of metabolite oxidation through the citrate cycle to adenosine triphosphate (ATP) production by the electron transport chain (ETC)^48^. Mitochondrial dysregulation leads to the disorders of energy metabolism and ETC, and the subsequent ROS burst^22^, which serves as important mediators in NLRP3 inflammasome activation^20^. In the present study, proteomics analysis discovered 63 proteins were reversed by RVS treatment in mice with CME. These 63 differentially expressed proteins mainly participated in the processes of citrate cycle, oxidative phosphorylation, respiratory chain complex and cellular response to redox state. SDH is an inner mitochondrial membrane protein complex that links the citrate cycle to the ETC. The most common implication of SDH in disease is its role in creating the environment favorable for excessive ROS production^49^. CME led to a significant increase in the expression of both SDHA and SDHB that paralleled mitochondrial disruption and ROS generation as demonstrated by TEM analysis and ROS detection, and these were inhibited by the addition of RVS. Given the close relationship between ROS and the activation of the NLRP3 inflammasome, we suspect that the anti-oxidant function may explain the mechanism of action of RVS to inhibit the NLRP3 inflammasome.

In conclusion, we uncover that the activation of NLRP3 inflammasome and myocardial pyroptosis contribute to cardiac injury following CME. Our findings unfold the role of NLRP3 inflammasome inhibitor in protecting against CME-induced cardiac injury, thereby shedding light on the potential value of targeting the NLRP3 inflammasome for treating CME. We also propose that RVS protects against CME-induced cardiac injury via alleviating ROS production and mitochondrial damage, consequently suppressing NLRP3 inflammasome activation, thus providing evidence for the clinical application of statins in preventing periprocedural myocardial injury and other CME-related diseases.

## Materials and methods

### Animal procedure and drug treatment

Adult male C57BL/6 mice (7–8-weeks old, 22–25 g) were obtained from LingChang BioTech Co. Ltd. (Shanghai, China). Mice were subjected to CME as described in our previous study^50^. Briefly, after the mice were anesthetized and ventilated with oxygen and 1.5% isoflurane (Baxter International Inc., Deerfield, IL, USA), a median thoracotomy was performed to expose the heart and ascending aorta. A total of 500,000 polyethylene microspheres (diameter 9 µm, Dyno Particles AS, Lillestrem, Norway) were injected into the left ventricle chamber during the occlusion of the ascending aorta for 15 s. The sham surgery mice received 0.9% saline instead of the microspheres in an equal volume. For the RVS treatment, mice were orally received 40 mg/kg/d RVS (AstraZeneca, Macclesfield, UK) three days before and after CME induction. To investigate the role of the NLRP3 inflammasome in CME-induced myocardial injury, another group of mice was injected intraperitoneally with a selective NLRP3 inflammasome inhibitor MCC950 (20 mg/kg/d, MedChemExpress, Princeton, NJ, USA) three days prior and after CME intervention.

### Echocardiography

Transthoracic echocardiography was performed using the Vevo770 instrument (Visual Sonics Inc., Toronto, Canada) to evaluate the cardiac function at 3 days after the establishment of CME. The mice were laid supine on a heated platform and anesthetized under continuous isoflurane flow at a rate of 1–2 L/min. LV end-systolic and end-diastolic volumes (ESV and EDV) were measured from M-mode echocardiogram of the parasternal long-axis view. LVEF was calculated from the following formula: (EDV − ESV)/EDV·100. FS was assessed on the basis of the percent changes of LV end-diastolic and end-systolic diameters.

### Histological and immunohistochemistry analysis

After echocardiographic examination, all mice were sacrificed by cervical dislocation under deep isoflurane anesthesia. Hearts were harvested, fixed in 4% paraformaldehyde, embedded in paraffin, and sectioned at 5 mm. Hematoxylin–eosin (HE) and Masson trichrome staining were used to exam cardiac morphology and collagen deposition respectively. To reveal the expression of NLRP3 inflammasome-related proteins, the sections were soaked in 3% hydrogen peroxide to quench endogenous peroxidase activity and then were incubated with anti-NLRP3, anti-IL-1β (Abcam, Cambridge, UK) at 4 °C overnight for immunohistochemistry. Microinfarct areas were stained with Heidenhain’s iron hematoxylin (Solarbio Life Sciences, Beijing, China) following the manufacturer’s instruction. Heidenhain’s iron hematoxylin staining is an important method for detecting early myocardial ischemia, wherein the ischemic myocardium is in dark gray color, while the normal myocardial cells were stained into red color^51^. Five vision fields (×200 magnification) of each section were randomly selected under an Olympus BX-51 light microscope (Olympus, Tokyo, Japan). The percentage of microinfarct size was calculated as the ratio of microinfarct area to the total evaluated area quantified by Image J software (Version 1.50, NIH, Bethesda, MD, USA).

### Immunofluorescent double staining

For immunofluorescent double staining of Troponin T and NLRP3, paraffin-embedded cardiac sections were deparaffinized, rehydrated, antigen retrieved, and then permeabilized with 0.1% Triton X-100 and 1% bovine serum albumin. Thereafter, the sections were incubated with anti-Troponin T (Abcam, Cambridge, UK) and anti-NLRP3 (Boster Biological Technology, Wuhan, China) overnight at 4 °C. Binding sites of the primary antibodies were revealed with Alexa Fluor 546 goat anti-mouse and Alexa Fluor 488 goat anti-rabbit secondary antibodies (Thermo Fisher Scientific, Waltham, MA, USA). The nuclei were stained with 4′,6-diamidino-2-phenylindole (DAPI; Sigma-Aldrich, St Louis, MO, USA). Images were captured using an Olympus IX51 fluorescence microscope (Olympus, Tokyo, Japan).

### Cell culture and treatments

H9c2 cardiomyoblasts were purchased from the Cell Bank of Type Culture Collection of Chinese Academy of Sciences and cultured with DMEM (Hyclone, Logan, UT, USA) supplemented with 10% fetal bovine serum (Hyclone, Logan, UT, USA) in a humidified atmosphere with 5% CO_2_ at 37 °C. To imitate the ischemic micro-environment induced by CME, the H9c2 cells were incubated with 40 ng/ml TNF-α (Peprotech, Rocky Hill, CT, USA) and exposed to hypoxia for 12 h. For the RVS intervention, the cells were pretreated with 20 µM RVS (Selleck Chemicals, Houston, TX, USA) for 2 h before the stimulation of TNF-α and hypoxia. To demonstrate the contribution of caspase-1 and ROS to the pyroptotic cell death, the caspase-1 selective inhibitor VX-765 (Selleck Chemicals, Houston, TX, USA) and ROS scavenger NAC (MedChemExpress, Princeton, NJ, USA) were added 2 h before the stimulation of TNF-α and hypoxia at 20 μM and 5 mM respectively.

### Measurement of caspase-1 activity

Cells with active caspase-1 were identified using the pyroptosis/caspase-1 assay kit (Immunochemistry Technologies, Bloomington, MN, USA) according to the manufacturer’s instruction. Shortly, the H9c2 cells were trypsinized to create suspensions, and then incubated with FLICA (FAM-YVAD-FMK) probe for 1 h at 37 °C. After three washes with washing buffer, the cells were resuspended in 200 μl PBS and analyzed immediately using a FACS Aria flow cytometer (BD Biosciences, San Jose, CA, USA).

### Cytotoxicity assay

Blood samples of the mice were collected from orbital sinus and centrifuged at 3000 rpm at 4 °C for 15 min to separate serum. The levels of LDH in serum and cell culture supernatants released from injured cardiomyocytes were determined by the LDH kit (Jiancheng Bioenginering Institute, Nanjing, China) following the manufacturer’s instruction. The absorbance was measured at a wavelength of 450 nm.

### Cell viability assay

The H9c2 cardiomyoblasts were seeded in 96-well plates at a density of 10^5^/ml. After aforementioned treatment, the cells were incubated with the CCK-8 assay reagent (Dojindo Laboratories, Kumamoto, Japan) following the manufacturer’s instruction. A microplate reader was used to measure the absorbance at 450 nm.

### Hoechst 33342/PI fluorescent staining

Hoechst 33342 and propidium iodide (PI) double staining was used to identify the pyroptotic H9c2 cells as described previously^12^. At the end of all interventions, the cells cultured in 6-well plates were fixed with 4% paraformaldehyde and then stained with 5 mg/ml Hoechst 33342 and 10 mg/ml PI (Beyotime Institute of Biotechnology, Shanghai, China) for 10 min in the dark. The stained images were observed using an Olympus IX51 fluorescence microscope (Olympus, Tokyo, Japan).

### Proteomics analysis

Equivalent heart samples (50 mg) from sham, CME and RVS-treated mice were homogenized to extract total proteins using a protein extraction kit (Thermo Fisher Scientific, Waltham, MA, USA). All samples were digested with trypsin and then labeled with tags using an iTRAQ reagent kit (AB Sciex, Foster City, CA, USA) following the manufacturer’s protocol. Labeled samples were separated using Easy nLC1000 system (Thermo Fisher Scientific, Waltham, MA, USA) and processed for gradient elution by a C18 analytical column (Thermo Fisher Scientific, Waltham, MA, USA). Liquid chromatography coupled with tandem mass spectrometry (LC-MS/MS) was performed using a Q-Exactive (Thermo Fisher Scientific, Waltham, MA, USA) mass spectrometer. The MS raw files were processed using Proteome Discoverer 1.3 software (Thermo Fisher Scientific, Waltham, MA, USA). For the bioinformatics analysis, differentially expressed proteins were defined as more than 1.2-fold change (≥1.2 or ≤0.83) and the *P* value < 0.05 among the groups. We performed the analysis of Gene Ontology (GO) enrichment, KEGG pathways and protein–protein interaction (PPI) network by using the online bioinformatics data analysis tool OmicsBean (http://www.omicsbean.cn).

### Transmission electron microscopy (TEM)

The cardiac tissues were fixed in 2.5% glutaraldehyde and 1% OsO4, then embedded in resin. Ultra-thin sections were counterstained with uranyl acetate and lead citrate and observed using a transmission electron microscope (Hitachi, Ibaraki, Japan).

### Detection of ROS generation

Mice ventricular tissues were embedded in OCT compound (Sakura Fine Technical, Tokyo, Japan) to freeze on liquid nitrogen and were cut into 5-μm-thick sections. The sections were incubated with 5-µM DHE fluorescence probe (Beyotime Institute of Biotechnology, Shanghai, China) for 30 min at 37 °C protected from light, followed by three 5-min washes in PBS. Fluorescence images were acquired by an Olympus IX51 fluorescence microscope (Olympus, Tokyo, Japan). In vitro, mitochondrial superoxide formation was detected by incubating cells in the dark with 5 µM MitoSOX red dye for 30 min at 37 °C and then washed with culture medium. Images were obtained with the FV3000 confocal laser scanning microscope (Olympus, Tokyo, Japan) at 510/580 nm. The MFI of ROS from five random fields of each sample was analyzed using Image J software (NIH, Bethesda, MD, USA).

### Western blotting

Proteins from ventricular tissue and H9c2 cells were isolated using RIPA lysis buffer (Beyotime Institute of Biotechnology, Shanghai, China) with protease and phosphatase inhibitors (Thermo Fisher Scientific, Waltham, MA, USA). Equal amounts of proteins (40 μg) were electrophoresed on 8–12% sodium dodecyl sulfate-polyacrylamide gel, and then electroblotted onto PVDF membranes (Millipore, Temecula, CA, USA). The membranes containing target proteins were blocked with 5% bovine serum albumin for 1 h at room temperature. Proteins were reacted using primary antibodies against NLRP3, IL-1β, SDHA, SDHB (Abcam, Cambridge, UK), caspase-1, GSDMD (Santa Cruz Biotech, Santa Cruz, CA, USA) and the loading control Vinculin (Sigma-Aldrich, St Louis, MO, USA) or GAPDH (Cell Signaling Technology, Danvers, MA, USA). The antigen-antibody complexes were incubated with HRP-conjugated secondary antibodies (Cell Signaling Technology, Danvers, MA, USA). The immunoblot images were acquired using an enhanced chemiluminescence kit (Thermo Fisher Scientific, Waltham, MA, USA) and a ChemiDoc system (Bio-Rad Laboratories, Redmond, WA, USA). Densitometric analysis was performed using Image Lab software (Bio-Rad Laboratories, Redmond, WA, USA).

### Statistical analysis

Data were expressed as mean ± SEM. Two-tailed Student’s *t* test was used to compare differences in two groups. For comparing ≥3 groups, differences were identified by one-way ANOVA followed by Tukey’s post-hoc comparisons. A *P* value < 0.05 was considered significant. Data were analyzed using GraphPad Prism version 5.0 (GraphPad Prism Software, La Jolla, CA, USA).

## Supplementary information

Supplementary figure legends

Figure S1
