# Supplementary material for: Rosuvastatin protects against coronary microembolization-induced cardiac injury via inhibiting NLRP3 inflammasome activation
Source: Cell Death Dis. 2021 Jan 12;12(1):78. doi: 10.1038/s41419-021-03389-1 (PMC7804109; doi:10.1038/s41419-021-03389-1)
Supplement: Supplementary file 1 — Supplementary figure legends [file 41419_2021_3389_MOESM1_ESM.docx]

**Supplementary Figure legend**

**Fig.S1** NLRP3 and IL-1β are upregulated in mice heart following CME. Representative images of immunohistochemical staining and integrated optical density (IOD) analysis of NLRP3 and IL-1β. Scale bars = 50 µm. Black arrows indicate the microspheres. n = 4 per group. The quantification is representative of the IOD in 5 fields ± SEM by random. ∗P < 0.05, ∗∗P < 0.01.
