# Supplementary figures and images for: Rosuvastatin protects against coronary microembolization-induced cardiac injury via inhibiting NLRP3 inflammasome activation
Source: Cell Death Dis. 2021 Jan 12;12(1):78. doi: 10.1038/s41419-021-03389-1 (PMC7804109; doi:10.1038/s41419-021-03389-1)

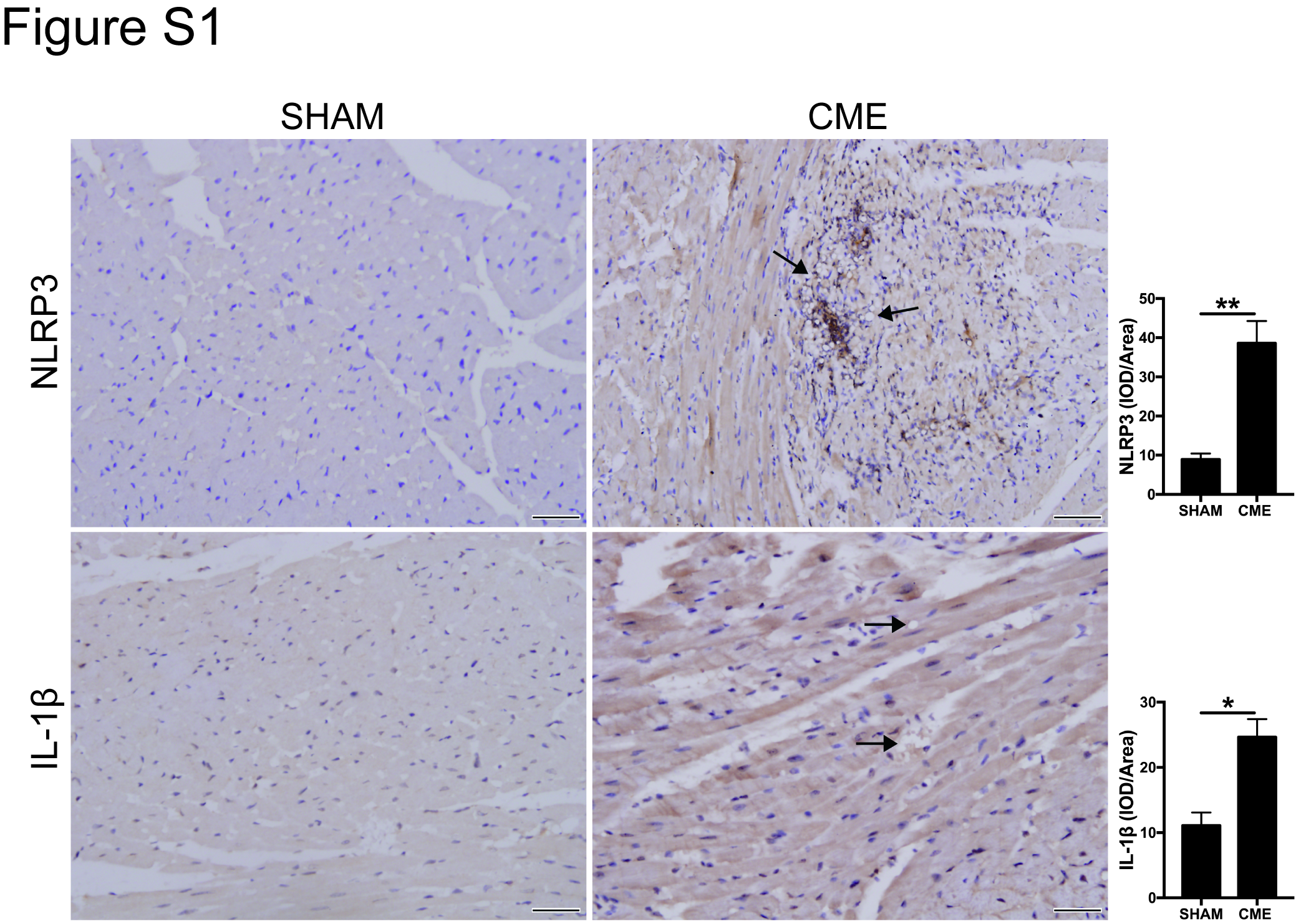

Supplement: Supplementary file 2 — Figure S1 [file 41419_2021_3389_MOESM2_ESM.tif]
